# Case Report: Diagnosis and treatment of *incontinentia pigmenti* with central nervous system anomalies in one patient

**DOI:** 10.3389/fped.2024.1490816

**Published:** 2025-01-15

**Authors:** Yun Li, Junbin Hong, Shangge Xu, Tong Zhou, Xiaolan Xiao, Jinghua Yang, Yiting Chen

**Affiliations:** Department of Pediatrics, The Second Affiliated Hospital, Guangzhou University of Chinese Medicine, Guangzhou, China

**Keywords:** *incontinentia pigmenti* (IP), case report, diagnosis, treatment, pathogenic mechanisms

## Abstract

**Introduction:**

This article reports a detailed case of a patient with *incontinentia pigmenti* who exhibited epileptic status and dermatologic symptoms.

**Case presentation:**

A 5-month-old female patient was brought to our hospital due to status epilepticus, with erythematous vesicular skin lesions on her trunk and extremities. Routine magnetic resonance imaging revealed infarction, ischemia, and encephalomalacia. Skin biopsy pathology indicated pigmentation disorder. Molecular genetic testing was conducted to identify IKBKG mutations, and the case was finally diagnosed with *incontinentia pigmenti* complicated by central nervous system anomalies. She was treated with oral levetiracetam (10 mg/kg/day, administered every 12 h) to control her recurrent seizures, and prednisone (1 mg/kg/day, once a day) for anti-inflammatory effects.

**Conclusion:**

After nine months, her skin lesions have resolved, with only a few newly developed erythematous papules and areas of hyperpigmentation being evident. There were no recurrent epilepsy symptoms, developmental impairments, or other associated symptoms.

## 1 Introduction

*Incontinentia pigmenti* (IP), also known as Bloch-Sulzberger syndrome, is a rare X-linked neurocutaneous disorder with a birth prevalence of 1.2 per 100,000 individuals (1). This disease is characterized by distinctive skin abnormalities in all patients and central nervous system (CNS) impairments in approximately one-third of all cases (2). IP arises from inherited mutations (10%–25% of patients) or sporadic *de novo* mutations (>75%) in the inhibitor of IKBKG (kappa B kinase gamma, previously known as NEMO) gene, located at Xq28 (3, 4). Skin abnormalities typically start as vesicobullous areas that morph into pigmented whorl-shaped lesions (5). While CNS impairments are less common than skin lesions, they significantly impact long-term prognosis and can lead to disabilities such as seizures, mental retardation, microcephaly, and hemiparesis (2, 6). Although the pathogenesis of CNS lesions remains unclear, high IKBKG expression in the CNS (7), along with inflammation, vascular abnormalities, and excessive apoptosis, have been proposed as potential underlying factors. Treatment options for IP remain controversial, with some reports suggesting the use of glucocorticoids for cerebrovascular lesions and others indicating that the lesions may resolve spontaneously without treatment (8). CNS abnormalities generally present in late infancy or early childhood (6). We recently treated a rare case of IP with CNS anomalies at 5 months of age, establishing therapeutic and long-term management strategies, along with a review of the complex pathophysiology of this disease.

## 2 Case presentation

### 2.1 General information

**Figure 1:**
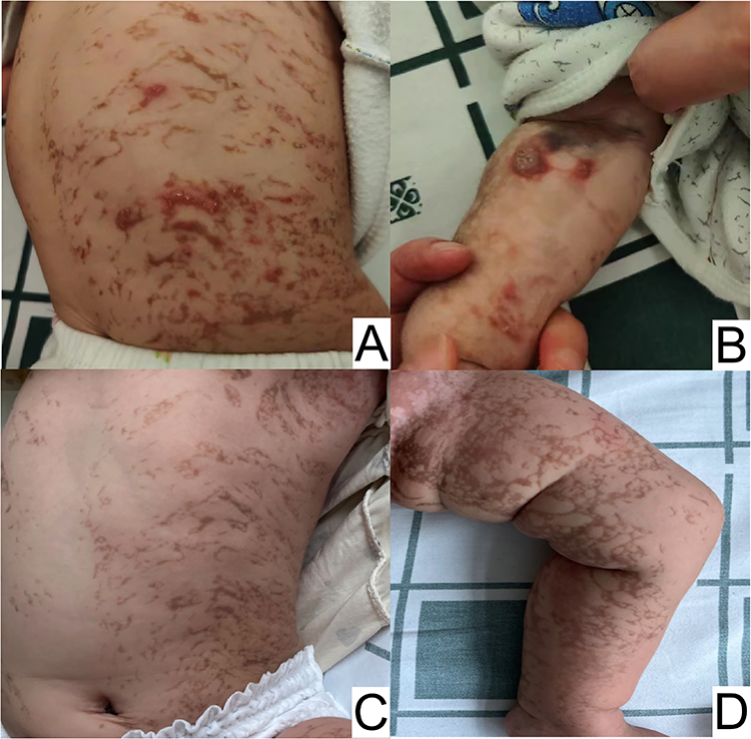
Overlapping skin lesions of erythema and blisters, verrucous hyperplasia, and pigmentation on the trunk **(A)**, and verrucous hyperplasia on the arm **(B)** at 5 months of age. Hyperpigmentation on the trunk **(C** and **D)** at 11 months of age.

A 5-month-old female patient was brought to our hospital due to status epilepticus. Five days prior, the patient had experienced general tonic-clonic seizures accompanied by eyeball deviation and loss of consciousness, lasting from several seconds to one minute, occurring once per day. Upon admission, she had developed recurrent clusters of brief twitches in her limbs and left side of her face for nearly 12 h. Her mother stated that the patient had manifested a distinctive rash characterized by erythematous papules and vesicles, which extended across her extremities and trunk. Throughout the following four months, the initial blistering manifestations subsided, and the characteristic skin alterations progressed to a subsequent phase, primarily exhibiting verrucous hyperplasia and hyperpigmentation. This progression underscores the dynamic nature of the dermatological condition and its adherence to the unique pattern defined by Blaschko's lines (Figure 1).

The child was born after a full-term gestation of 39 weeks, weighing a healthy 3,300 grams at birth. Her mother had also experienced similar skin lesions in childhood, manifesting as hyperpigmentation across the entire body. Currently, however, only a small area of hypopigmentation remains on the inner side of her left forearm. The mother reported no history of exposure to radioactive substances during pregnancy and no history of medication use. She also stated that the patient's brother and other family members are healthy, with no history of genetic disease.

### 2.2 Clinical findings

The patient appeared drowsy, with erythematous vesicular skin lesions on her trunk and extremities, but no CNS abnormalities or other systemic physical impairments were detected.

### 2.3 Diagnostic assessment

**Figure 2:**
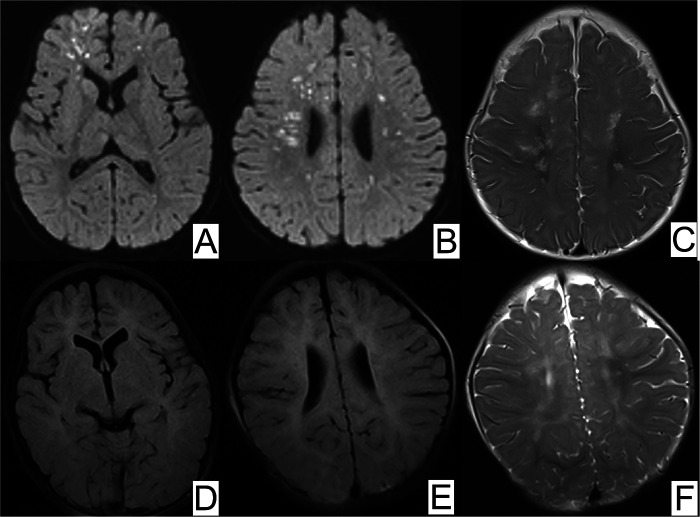
MRI of brain ischemia. **(A,B)** Magnetic resonance diffusion-weighted imaging (DWI) showed scattered foci of restricted diffusion in the bilateral frontal parietal occipital lobes and bilateral temporal lobes, **(C)** T2-weighted magnetic resonance image (MRI) displayed periventricular white matter hypersignal at 5 months old. At 11 months old, repeated DWI **(D,E)** showed normal signal and **(F)** T2-weighted MRI displayed slightly hypersignal in bilateral semiovale centers.

Laboratory analyses revealed elevated levels of tumor necrosis factor *α* (TNF-α) and a slightly higher ratio of eosinophils. Electroencephalography (EEG) revealed epileptiform discharges prominently in the right anterior temporal area, right middle temporal area, and right frontal area. Routine magnetic resonance imaging (MRI) revealed infarction, ischemia, and encephalomalacia. Magnetic resonance angiography (MRA) revealed the presence of anterior and right posterior communicating arteries, absence of the left posterior communicating artery, and tortuosity of the basilar arteries and intracranial segments of the bilateral vertebral arteries, all of which are considered developmental abnormalities (Figure 2). Echocardiography suggested a suspected patent foramen ovale (left-to-right shunt).

**Figure 3:**
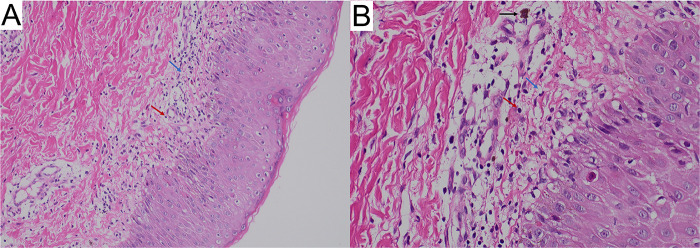
Skin biopsy showing inflammatory stage lesions typical of *incontinentia pigmentia* (hematoxylin-eosin; **(A)** original magnification ×100 and **(B)** original magnification ×400). The white arrows indicate vacuolar degeneration, red arrows indicate dermal inflammatory infiltrate including lymphocytes; black arrows, chromophilic cells.

**Figure 4:**
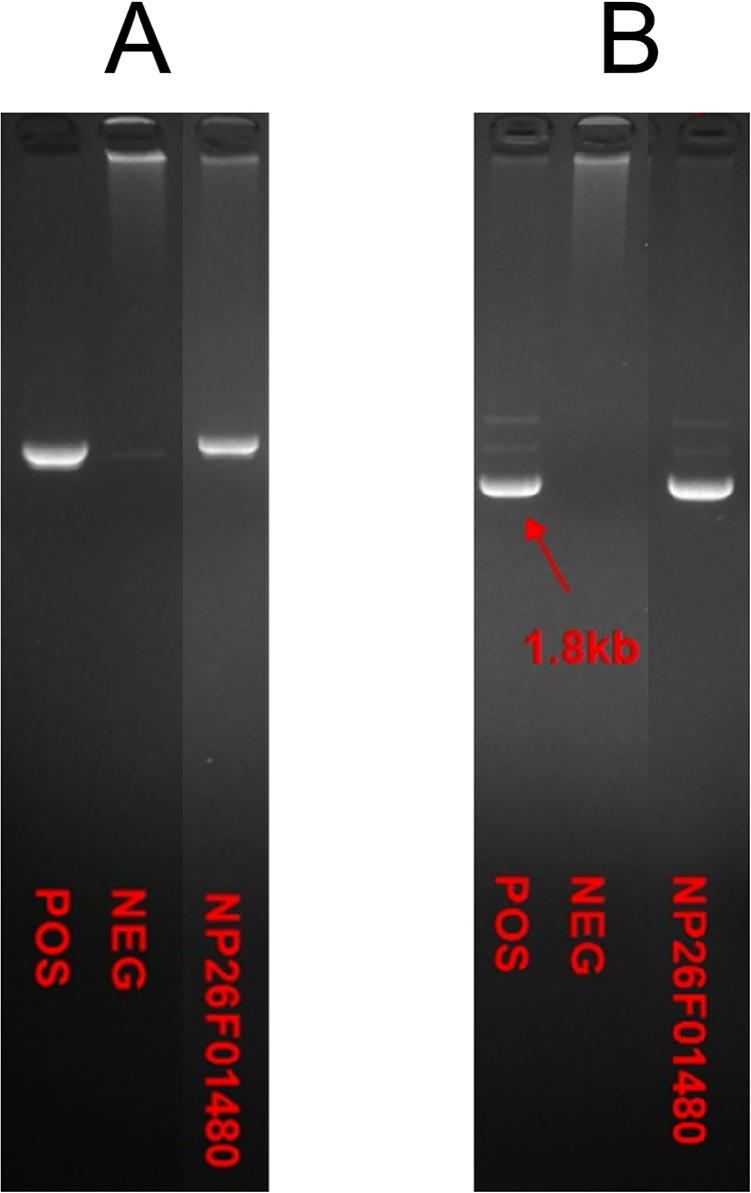
PCR analysis of IKBKG gene by agarose gel electrophoresis indicated the deletion of exon in IKBKG of this patient. The samples from left to right were positive control, negative control and the case respectively in **(A,B)**.

The skin biopsy revealed vacuolar liquefaction of the basal layer (of focal epidermis) accompanied by mild eosinophilic and lymphocytic infiltration in the superficial dermis, indicative of pigmentation (Figure 3). Molecular genetic testing utilizing high-throughput sequencing was conducted to identify IKBKG mutations. A 2.9 kb deletion on chromosome Xq28, spanning exons 4–10 of the IKBKG gene, was detected. This was confirmed by polymerase chain reaction (PCR) analysis of IKBKG gene (Figure 4). This IP case complicated with CNS anomalies was diagnosed using updated IP diagnostic criteria (6) and confirmed through molecular analysis of IKBKG (4).

### 2.4 Therapeutic intervention

The patient was treated with oral levetiracetam (10 mg/kg/day, administered every 12 h) to control her recurrent seizures. Additionally, a topical steroid cream was prescribed to manage the skin symptomology and prednisone (1 mg/kg/day, once a day) was administered for anti-inflammatory effects and to prevent further CNS anomalies.

### 2.5 Follow-up and outcomes

Upon discharge, the patient's caretakers were instructed to bring her to our hospital once a month. To date, she has not experienced any further seizures, and her skin lesions have resolved, with only a few newly developed erythematous papules and areas of hyperpigmentation being evident. Her developmental milestones were appropriate: at 14 months old, she was able to walk independently, crawl, pick up small objects, and babble single syllables. An MRI of the brain conducted at 11 months of age showed obvious improvement in the previously prominent lesions, and EEG demonstrated improved signs with single epileptiform discharges. A fundus examination revealed normal results, with deciduous teeth erupting at 8 months of age and no anomalies observed in her nails or breasts.

## 3 Discussion

IP is a rare neuroectodermal dysplasia caused by mutations in the IKBKG gene, located at Xq28. This gene spans approximately 33 kb and contains 10 exons and 9 introns. Our case involved deletions of exons 4–10 of the IKBKG gene, a mutation reported in 90% of IP cases (9). The IKBKG protein activates the nuclear factor kappa B (NF-*κ*B) signaling pathway, which plays a critical role in various key cellular processes, including proliferation, inflammation, cellular stress response, innate immunity, and protection against apoptosis (10). This mutation results in either partial or complete loss of function of NF-*κ*B, leading to increased susceptibility to apoptosis via activation of tumor necrosis factor *α* (TNF-α) and subsequent cell death (11).

IP cases typically exhibit characteristic skin lesions evolving through a sequential four-stage process along the Blaschko lines, starting with a vesiculobullous eruption (Stage I), progressing to a verrucous stage (Stage II), a hyperpigmented stage (Stage III), and culminating in an atrophic, hypopigmented stage (Stage IV) (4). At birth (or sometimes prenatally), wild-type NF-*κ*B cells and NF-*κ*B mutant cells compete on the skin surface of IP patients, specifically along the Blaschko lines. The mutant skin cells are eliminated by the wild-type cells via apoptosis (12), initiating the primary inflammatory, vesicular stage of skin changes. In the absence of normal NF-*κ*B activity, endothelial cells overexpress chemotactic factors, attracting eosinophils (13), which undergo degranulation and release proteases (14), leading to inflammation in the epidermis and other bodily areas. This results in intracellular edema (spongiosis) and ultimately blistering, characteristic of the disorder at birth (13).

In our patient, serum eosinophil levels were slightly elevated at admission, potentially due to the test being conducted at 5 months of age when her skin lesions were nearly verrucous and progressing towards hyperpigmentation. It is worth noting that multiple lesion types may coexist in different stages, and lesion location can vary in a stage-dependent manner (3). Our case initially presented with erythema and blisters, progressing to three distinct lesion types simultaneously at 5 months.

CNS abnormalities occur in 25%–50% of IP patients, most commonly in the first year of life and being more prevalent in male infants (15, 16). Early infantile acute encephalopathy is a classic feature of IP in patients with neurologic manifestations. Epilepsy is the most common symptom, present in 13%–25% of patients with IP, and typically occurs within the first week of life (17). Approximately 30% of children with IP will develop motor impairment, intellectual disability, and learning difficulties (1). Our patient experienced status epilepticus at 5 months of age without showing any developmental impairment at 14 months.

Brain imaging in IP patients often reveals ischemia, hemorrhage, and lesions predominantly in the subcortical white matter, causing severe neurological symptoms and infarction/hemorrhage. These abnormalities can also affect the corpus callosum, basal ganglia, and thalamus, but rarely the cerebellum and brainstem (2). Our case exhibited a similar pattern, with a notable lack of collateral vessels, unlike moyamoya disease. Prior studies suggest that prenatal arteriopathic changes in IP are due to reduced antiapoptotic gene transcription (18). MRA analysis in our patient revealed developmental abnormalities, consistent with antenatal cerebral pathology in IP (19).

The pathogenesis of CNS involvement in IP patients remains unclear, likely encompassing multiple pathologies. Studies suggest that neurological manifestations stem from IKBKG mutations, triggering endothelial cell death, microvascular rarefaction, cerebral hypoperfusion, and blood-brain barrier disruption (20). This disruption may favor proconvulsive factors, explaining seizures unrelated to ischemic stroke (21). A previous study in mouse models revealed the occurrence of vascular endothelial abnormalities due to the death of endothelial cells and disruption of the blood-brain barrier (20). Inflammation plays a significant role in IP, with pro-inflammatory cytokines such as TNF-α, interleukin-1(IL-1), and interferon-*γ*(IFN-*γ*) produced by activated NF-*κ*B contributing to cerebral vessel disease (9, 21). TNF-α also attracts eosinophils, which can cause inflammation, vascular occlusion, and ischemia in the brain, similar to what is observed in skin lesions (22). Our patient exhibited elevated TNF-α levels. Random X-inactivation may underlie severe CNS abnormalities, while skewed inactivation may mitigate severity (23). IKBKG exon 4–10 deletions are linked to CNS abnormalities (24). However, CNS anomalies in IP patients likely derive from multiple gene mutations, all of which could contribute to the phenotypic spectrum.

Early IP diagnosis is crucial, as some patients may have normal neonatal neuroimaging but develop abnormalities later. Next-generation sequencing (NGS)/whole exome sequence (WES) pipeline analysis constitutes a powerful tool for accelerating IKBKG mutational screening (25). Additionally, long-term neuroimaging and advanced imaging techniques are important for monitoring disease progression. When necessary, anti-epileptic and/or corticosteroid treatment should be administered (1). In the case presented in our report, the patient was treated with levetiracetam oral solution (the dosage was initially 10 mg/kg/day, administered orally once every 12 h for a period of 6 months. Subsequently, it was reduced to 5 mg/kg/day, also administered orally once every 12 h, and has been continued to the present time) and prednisone (a dosage of 1 mg/kg once daily for one month, followed by a gradual tapering of the dose: 0.5 mg/kg once daily for one month, 0.25 mg/kg once daily for 15 days, 0.25 mg/kg once every other day for 15 days, and then increasing the number of days between doses every 15 days until complete withdrawal at six months). During the nine-month follow-up visits, the patient did not experience any recurrent spasms, and her developmental milestones were appropriate. Brain imaging conducted after six months of treatment revealed significant improvement. Studies have reported that high-dose corticosteroid therapy is effective in managing the neonatal dermopathy and encephalopathy associated with IP (26). Immunosuppressive and neuroprotective therapies may help prevent the exacerbation of arteriopathy, although steroid pulse therapy combined with heparinization should be cautiously considered in patients with intracranial hemorrhage (27). Chan et al. described a tightly regulated steroid control of the NF-*κ*B pathway in brain microglia (28). Furthermore, reducing TNF-α activity may represent a potential treatment strategy to prevent cerebral ischemia, possibly through its effects on NF-*κ*B (26). Gene therapy is currently being investigated for its potential to correct severe cerebrovascular pathology (29).

Neurological sequelae are frequently observed in the CNS of IP patients, necessitating early management by a rehabilitation team to mitigate neurocognitive and/or orthopedic complications. Although no definitive treatment exists, early intervention is strongly recommended. Fortunately, our patient has not experienced any neurological sequelae; however, ongoing follow-up with neurology departments remains crucial. Additionally, a long-term, multidisciplinary approach is vital for predicting potential complications that may arise later in life and for better managing IP. We provided her parents with a comprehensive list of all scheduled appointments, and advised the foster family that adhering to these appointments was an essential first step in her care plan.

## Data Availability

The raw data supporting the conclusions of this article will be made available by the authors, without undue reservation.
